# The Promise of Intravaginal Rings for Prevention: User Perceptions of Biomechanical Properties and Implications for Prevention Product Development

**DOI:** 10.1371/journal.pone.0145642

**Published:** 2015-12-22

**Authors:** Kate Morrow Guthrie, Sara Vargas, Julia G. Shaw, Rochelle K. Rosen, Jacob J. van den Berg, Patrick F. Kiser, Karen Buckheit, Dana Bregman, Lara Thompson, Kathleen Jensen, Todd Johnson, Robert W. Buckheit

**Affiliations:** 1 Centers for Behavioral & Preventive Medicine, The Miriam Hospital, Providence, Rhode Island, United States of America; 2 Department of Psychiatry & Human Behavior, The Warren Alpert Medical School, Brown University, Providence, Rhode Island, United States of America; 3 Department of Behavioral & Social Sciences, Brown University School of Public Health, Providence, Rhode Island, United States of America; 4 Department of Bioengineering, University of Utah, Salt Lake City, Utah, United States of America; 5 ImQuest BioSciences, Frederick, Maryland, United States of America; Commissariat a l'Energie Atomique(cea), FRANCE

## Abstract

Intravaginal rings (IVRs) are currently under investigation as devices for the delivery of agents to protect against the sexual transmission of HIV and STIs, as well as pregnancy. To assist product developers in creating highly acceptable rings, we sought to identify characteristics that intravaginal ring users consider when making decisions about ring use or non-use. We conducted four semi-structured focus groups with 21 women (aged 18–45) who reported using an IVR in the past 12 months. Participants manipulated four prototype rings in their hands, discussed ring materials, dimensionality, and “behavior,” and shared perceptions and appraisals. Five salient ring characteristics were identified: 1) appearance of the rings’ surfaces, 2) tactile sensations of the cylinder material, 3) materials properties, 4) diameter of the cylinder, and 5) ring circumference. Pliability (or flexibility) was generally considered the most important mechanical property. Several ring properties (e.g., porousness, dimensionality) were associated with perceptions of efficacy. Women also revealed user behaviors that may impact the effectiveness of certain drugs, such as removing, rinsing and re-inserting the ring while bathing, and removing the ring during sexual encounters. As product developers explore IVRs as prevention delivery systems, it is critical to balance product materials and dimensions with use parameters to optimize drug delivery *and* the user experience. It is also critical to consider how user behaviors (e.g., removing the ring) might impact drug delivery.

## Introduction

Women and girls are disproportionately affected by the HIV/AIDS epidemic, comprising about 57% of the estimated 25 million people living with HIV/AIDS in sub-Saharan Africa and approximately half of the global HIV/AIDS burden [1]. The impact of sexually transmitted infections other than HIV is also alarming, with the greatest burden faced by women in developing countries [2]. In addition to the burden of disease, the family planning needs of women across the globe far exceed adequate supply of, and access to, affordable contraceptive options [3]. Prevention methods that can be accessed and used effectively by women are urgently needed.

Intravaginal rings (IVRs) are emerging as potential devices for delivery of microbicides to protect women against HIV infection, and potentially ameliorate other sexual and reproductive health concerns. IVRs have a number of advantages over other prevention methods: they can be user-controlled, are potentially less detectable to male partners than topically applied peri-coital gel formulations, do not require daily or coitally-associated application [4], and can provide protection over long periods of time. Importantly, it may be possible to use IVRs to deliver more than one prevention active pharmaceutical ingredient (API), a concept known as multi-purpose technologies (MPT). This could include APIs to prevent other sexually transmitted infections, or contraceptive agents [5].

Several IVRs are currently on the market for contraceptive and hormone replacement purposes [6]. IVRs for delivery of microbicides to prevent vaginal HIV transmission are now in development and under evaluation. Phase I and II trials have demonstrated the safe and effective delivery of one anti-HIV microbicide in an IVR, and two efficacy studies are underway [7–9]. The completion and success of such a trial depends upon a number of factors, including maintaining product quality during large-scale production, minimizing the cost of materials and manufacturing, and designing an IVR that is feasible for long-term use [10–12].

Beyond feasibility, however, if a biomedical prevention strategy such as a microbicide is to be effective for HIV prevention, it must also be sufficiently acceptable to women, such that they are willing to use it consistently for extended periods of time. Phase III efficacy trials of HIV prevention microbicides delivered via vaginal gels have suffered from poor adherence to product use, which is likely, at least in part, affected by the user’s experience of the product during use [13–15]. The acceptability data from contraceptive and sexual health IVR technology have shown promising results, although acceptability has mainly been studied in the context of clinical trials [16–18] and clinical evaluation programs [19,20]. Within these studies, acceptability is generally assumed to correspond to continuation rate [16,18,21,22], or is assessed via questionnaire after several cycles of use [20,23,24]. Data from these trials suggest that most users find IVRs acceptable [25] despite reports of involuntary IVR expulsion [22,26] and interference with sexual intercourse [21,27,28].

Few in-depth (i.e., qualitative or mixed methods) studies have investigated the acceptability of IVRs among IVR-experienced or IVR-naïve women. Results from published studies highlight concerns related to IVR use, including the need for provider-facilitated education and counseling regarding insertion and removal, user concerns about feeling the IVR during intercourse, as well as during the course of the user’s daily activities, and the impact of IVRs on hygiene (particularly with long-term use [29–31]). The majority of acceptability data comes from women using IVRs in 28-day cycles (21 days of IVR use followed by 7 days without); however, a few studies have examined extended use regimens. Women using IVRs continuously for an extended period of time have found the devices generally acceptable, with most discontinuations due to menstrual irregularities secondary to IVR use for contraception [32–34]. In a study among Chilean women who used IVRs continuously for 3 to 14 months during breastfeeding instances of discontinuation were attributed to frequent expulsion, excessive vaginal discharge, interference with sex life, and user responsibility for insertion and removal (in comparison to physician insertion and removal, as with an IUD). Nonetheless, most women in the study reported an overall positive experience related to IVR use, and some who had discontinued IVR use expressed potential future interest in IVR use if their specific issues could be effectively addressed [31].

These preliminary explorations of acceptability are especially encouraging in light of research that suggests that an IVR releasing an anti-HIV microbicide is feasible [10] and potentially acceptable in areas where HIV is highly prevalent [30]. However, very little research has been conducted on IVR design preferences among women or their sex partners. Some female sex workers who handled a prototype IVR during a focus group study in urban Kenya expressed concerns that the IVR was too big (both overall ring circumference and cylinder diameter) and that the cylinder was too hard. Both male and female focus group participants in the study expressed concern about the device’s impact on male sexual pleasure [30]. While these data cannot assure prediction of rates of actual use, potential users’ first impressions of products can critically impact whether they try and/or continue with initial use and product adoption.

Early incorporation of user experiences and preferences in IVR design is critical, as parameters including size, material composition, and mechanical properties will likely directly affect acceptability and adherence [6], both initially and over time. Investigations of product properties are well-established in the food and cosmetic industries, but have not, until recently, been incorporated into the development of HIV prevention products or MPTs[35]. Studies are needed to systematically evaluate and measure users’ sensory perceptions and experiences of sexual health products to better understand the relationships between product properties and use patterns. Evaluation tools (i.e., Perceptibility Scales) used to objectively measure user sensory perceptions and experiences (USPE) of formulation and/or device characteristics and their performance during use, including sexual intercourse, are being developed to study properties of vaginal and rectal products (e.g., gels, films, and rings) that are most salient to users [36,37].

This pro-active, user-centered approach to prevention product design will facilitate success in future clinical trials and prevent the unnecessary use of resources on the development and clinical assessment of devices with little prospect of real-world acceptance. An IVR with an optimized user experience will be used by more women, more consistently, and as directed, therefore having the greatest possible impact on the HIV epidemic—or, in the case of MPTs, on a broader range of sexual and reproductive health conditions.

The current study explored the interaction of IVR materials, dimensionality (size/diameter), and biomechanical performance of four prototype IVRs among current and previous contraceptive ring users to more fully investigate the specific user sensory perceptions and experiences that might be relevant for the design of microbicide and multi-purpose technology IVRs. Our findings may be able to assist product developers in exploring the most salient factors that users consider in decision-making with respect to IVR use.

## Methods

### Participants

This formative qualitative work elicited the input of vaginal ring users, informed by their own product use experiences, to provide insight into the user sensory perceptions and experiences (USPE) most relevant for preclinical product design and iteration. The preclinical context imposes limitations on human subject research, minimizing sample sizes and activities to ensure subject safety. Participants were recruited from cities in the northeastern United States via community-based organizations, advertisements (print and online), and word of mouth. Recruitment materials invited vaginal ring users to attend a focus group to provide researchers with their opinions about how to design products women will want to use.

To be eligible for the study, women must have: 1) been 18–45 years old, 2) reported a regular menstrual cycle of 24–34 days, 3) reported vaginal sex with a man in the past 12 months, and 4) reported having used an intravaginal ring (IVR) within the last 12 months (i.e., all were recent or current contraceptive ring users). Women were ineligible if they were pregnant or breastfeeding to avoid any potential confounds with respect to motivations to use prevention products. Because participants would be manually manipulating prototype placebo rings in their hands during the focus group, women were also excluded for safety reasons if, upon inspection, they were observed to have signs of palmar or digital trauma, such as abrasions, cuts, or burns, as well as any dermatological conditions extending to their hands. All participants completed a brief sexual/reproductive health history and demographic survey prior to the start of the focus group discussion. Table 1 presents selected demographic characteristics for enrolled participants. IRB approval was granted by Lifespan/The Miriam Hospital Institutional Review Board (FWA00003538) and Western IRB (IRB00000533). All participants provided written informed consent.

**Table 1:** Selected Demographic Characteristics of Sample.

Product:	Ring (N = 21: *k* = 4)	
**Age**	**Mean**	**SD**
	28.2	6.0
	**n**	**%**
**Ethnicity**		
Hispanic/Latina	1	4.8%
**Race** ^a^		
Black/African American	2	9.5%
Caucasian/White	14	66.7%
Asian	3	14.3%
Multiracial	1	4.7%
Other or Do not identify by race	1	4.8%
**Education**		
Beyond high school	21	100.0%
**Current Marital Status** ^b^		
Never been married	13	61.9%
Married	7	33.3%
Divorced	1	4.8%
**Household Income (annual)**		
<$15,000	3	14.3%
$15,000-$35,999	6	28.6%
>$36,000	11	52.4%
Not reported	1	4.8%
**Sexual History**		
Oral sex, past 12 months	18	85.7%
Anal sex, past 12 months	5	23.8%
**Vaginal Product History**		
Vaginal medication	17	81%
Vaginal douche	3	14.3%
Vaginal lubricants	16	76.2%
Spermicides	2	9.52%
Desiccants	1	4.8%
Intravaginal rings^c^	21	100.0%
**Number of vaginal deliveries**		
0 (none)	17	81.0%
1 or more	4	19.0%

^a^: None of the participants reported “American Indian/Alaska Native” or “Native Hawaiian/Pacific Islander” as a racial identity.

^b^: None of the participants reported being legally “separated”

^c^: Inclusion Criterion

### Data Collection

Focus groups were conducted in the context of a preclinical topical microbicide drug development program (NIH: U19AI077289). A semi-structured focus group agenda was used to guide discussion in each group. Groups were facilitated by a doctoral-level qualitative methodologist with extensive experience in user-centered microbicide evaluation and acceptability. Note-takers were assigned 2–3 participants to observe throughout the discussion, to capture nonverbal communications and describe/illustrate ring manipulations performed by participants. The focus group was designed to capitalize on participants’ previous experience with intravaginal rings, and to elicit their perceptions of an HIV preventive microbicide IVR grounded in that prior experience. Participants were asked to discuss several topics of interest related to IVRs: 1) perceptions of the IVR insertion process, 2) conditions in which an IVR might come out of the vagina (e.g., involuntary vaginal ring expulsion, voluntary removal and replacement), 3) experiences of physical awareness of the IVRs they have used, 4) thoughts on the potential effects of prototype IVRs on the sexual experience, 5) thoughts about the prototype IVRs’ capacity to be used covertly, and 6) the “meaning” and “felt experience” of having IVRs in their bodies for extended periods of time.

Participants first discussed their likes and dislikes of the IVR they had experienced, including reasons for stopping use (if applicable), and their comfort (e.g., with application, in their daily activities, and during intercourse). This discussion served as the foundation for exploring participant concepts and attributes communicated during examination and manipulation of the prototype rings.

The prototype rings differed in material and cylinder diameter: Ring A (4 mm cylinder diameter made of Tecoflex EG-85A); Ring B (5 mm cylinder diameter made of Tecoflex EG-85A); Ring C (4 mm cylinder diameter made of Tecoflex EG-93A); and, Ring D (5 mm cylinder diameter made of Tecoflex EG-93A). The EG-93A is considered stiffer than the EG-85A, as measured by the Young’s modulus (a measure of the stiffness of materials). Since the force to compress the IVR is proportional to diameter of the cylinder, a small increase in diameter (e.g., 4 mm and 5 mm), with material modulus kept constant, can result in a large increase in the overall stiffness of the ring as an object and hence the force needed to compress the ring. Conversely, one could vary the force to compress by altering the modulus using different materials, but holding the cylinder diameter constant.

These variables were used to systematically explore the impact of dimensional and materials properties of the rings on user perceptions and opinions, including user perceptions of material hardness, flexibility, strength, and size (both overall and with respect to cylinder diameter). In similar fashion, device design parameters could be used to vary texture of the ring surface from both a tactile and visual perspective. The color and surface reflectivity of the ring material can also be varied as a function of ring structure (e.g., reservoir versus matrix ring) and its “interaction” with the active ingredients within.

The prototypes were specifically designed for use in this study, to represent a range of properties that may be experienced by the user and encourage discussion (e.g., softer versus harder material), and were not intended to represent any IVRs that are currently available or under investigation. The key research question was to identify those IVR properties that are most salient to potential users and better understand the feasible and acceptable range of properties with which they might be comfortable.

### Data Management and Analyses

Each focus group lasted for approximately 2 hours, inclusive of the consent process and survey completion. Discussions were audio recorded and transcribed verbatim. Transcripts were then reviewed by staff for completeness and cleaned (i.e., transcriptionist omissions and “unintelligibles” reviewed and addressed, personal health information obscured). A coding structure was developed, first as a function of the focus group agenda and research questions (see above), then, iteratively as concepts were identified or refined. Transcripts were independently coded by two doctoral-level researchers. Codes were discussed, discrepancies clarified, and final codes entered into NVivo qualitative data management software. Major constructs were summarized for preliminary results. Greater depth in analyses was achieved using comparative and thematic analyses strategies [38].

## Results

Four semi-structured focus groups were conducted with a total of 21 women in the greater metropolitan areas of southeast New England. Data analyses suggest that five salient characteristics of the prototype intravaginal rings influenced women’s anticipated experiences in using IVRs for HIV prevention. These included the appearance of the surface of the cylinder material, the tactile sensations of the surface of the cylinder material, the perceived materials properties, the prototype rings’ dimensionality (i.e., cylinder diameter, outer diameter), and the interaction of these elements. Each was evaluated with respect to its impact on product use parameters. Women often used their experience with the contraceptive ring as a referent. Given the sample studied, the ring referenced was the NuvaRing^®^, described as “a non-biodegradable, flexible, transparent, colorless to almost colorless,… [polymeric ring] with an outer diameter of 54 mm and a cross-sectional [cylinder] diameter of 4 mm” [39]. Overall, the majority of participants noted that Rings A and B were most like the intravaginal ring with respect to material and dimensional properties. Relevant quotes are provided in Table 2.

**Table 2:** Summary Participant-derived Characteristics, Primary Attributions, and Selected Illustrative Participant Quotes by Ring Characteristic.

	**SURFACE APPEARANCE**
**Matte/Opaque**	• Looks easy to squeeze/hold for insertion
	• Looks porous, to facilitate drug delivery
**Shiny**	• Looks slippery
	• Looks likely to be dropped during insertion
	• Looks prone to involuntary expulsion
**Illustrative Quotes**	*The things that I have experienced that are opaque are less slippery than things that are shiny*. [ID#37, G1]
	*…*, *it’s almost like*, *a little more porous*. *Like when I touch it*, *I can picture the hormones*, *you know*, *coming out of it on contact with my body…* [ID#46, G4]
	*I think it’s the shininess for me that…makes me think of plastic and when you think of plastic*, *I think of things that are*, *like*, *not as porous*. [ID#48, G4]
	**TACTILE SENSATIONS**
**Textured**	• Easy to squeeze/grip for insertion
	• Feels porous, to facilitate drug delivery
	• Consistent with a medical purpose
**Smooth**	• Feels slippery, oily
	• Feels industrial
	• Feels like that penis would glide over during sex
**Illustrative Quotes**	*It feels a little oily or slippery*, *it feels like it would be hard to hold on to when you’re trying to insert it*. [ID#43, G3]
	*There’s enough … friction on it when you rub it with your finger that … it wouldn’t slip out as easily if you were concerned about that*. [ID#42, G4]
	*… Perceptually*, *because of the matte feel of the [contraceptive ring]*, *I feel confident that … it’s leaching contraception*. *Because of the glossy finish on this*, *…I don’t react to it as though it’s going to leach*. [ID#40, G3]
	**MATERIALS PROPERTIES**
**Soft**	• Pliable
	• Easy to squeeze
	• Likely comfortable in vagina
	• Likely less noticeable in vagina (both daily use and sexual intercourse)
	• Less substantive with respect to long-term residence (sustained release)
**Hard**	• Likely to feel stiff in vagina
	• Likely more noticeable in vagina
	• Likely to feel like a physical barrier during sex
	• More durable for long-term residence (sustained release)
**Illustrative Quotes**	*[The prototype ring] looks like it’s more difficult to hold and maintain the shape*, *to get it into a shape of which to insert it into your vagina*. [ID#40, G3]
	*Since it’s harder to squeeze it makes me feel like it’s kind of stiffer and less flexible so when it goes in*, *are you gonna feel it*, *‘cause it’s not really gonna relax as much as per say the [contraceptive ring]*. [ID#30, G2]
	*I think it would be hard to find a comfortable position for it… Just because it’s so stiff and it wants to hold that round shape* [ID#43, G3]
	*… it’s too ‘not flexible*,*’ it … seems like it would be …irritating*. *Like*, *it would chafe*. [ID#34, G2]
	**DIMENSIONALITY**
**Smaller Cylinder Diameter**	• Easy to handle for insertion
	• Likely more comfortable in vagina
**Larger Cylinder Diameter**	• Likely very noticeable
	• Possible obstruction to penis during sex
	• Potentially uncomfortable during sex
	• Could hold more drug for long-term residence (vs smaller diameter)
**Illustrative Quotes**	*… it’s gonna make the experience of insertion feel like a long time ‘cause you’re gonna really feel it traveling*, *‘cause it’s so thick*. *And that is bad*, *in case you were wondering*. [ID#30, G2]
	*My husband would complain about…it bothering with the super sensitive underside*, *like*, *where the head meets the shaft of the penis*, *it would get caught on the bottom of the ring as it comes out*. [ID#46, G4]
	*… I wouldn’t be opposed to having it be a bit bigger [overall ring diameter] if it was thinner [cylinder diameter]…* [ID#44, G3]
	**INTERACTIONS OF MATERIALS AND DIMENSIONALITY**
**Illustrative Quotes**	*… I would be willing to insert the bigger [cylinder] if it was more pliable…I feel like pliability is probably more important to me than [cylinder] diameter*. [ID#45, G4]
	*And you can’t twist this one into a folded 8*. *Well*, *you can*, *but it’s like you have to really hold it*. *It’s just not as flexible*. *It’s too thick*. [ID#31, G1]
	*Yeah*, *the spring back*, *not that it’s going to be like earth shattering*, *but you might … feel the—dink [sensation of feeling the ring spring back into shape]—a little bit*. [ID#30, G2]
	*For me*, *if I’m using it for HIV prevention*, *which is paramount*, *I would want it to spring back because I’d feel like it was more secure if it was all the way open—*[ID#37, G1]
	**CONVENTIONAL ACCEPTABILITY ELEMENTS**
**Illustrative Quotes**	*I’m definitely for the see-through… I can see the liquid*, *hormones*, *I like that…*. *This looks like it’s gonna do what it’s gonna do*. [ID#30, G2]
	*I do prefer the… non-color in a way*, *but I can see the benefit of … it being white…if you thought that it might be dirty … and you washed it*, *… being able to … see that it’s not dirty*. [ID#48, G4]
	*It would be convenient if there was a ring that started out as white*, *and then went clear*, *then you knew that it was like running out of hormones or something*. [ID#41, G3]

### Ring Surface Appearance

Participants were asked to consider the appearance of the prototype ring surfaces. Perceptions and discussions ranged from a “matte” finish to a “shiny” finish. Women anticipated that a matte finish (compared to the shiny finish) would be easier to hold during insertion and, in particular, would allow the user to have a better hold on the ring upon squeezing in preparation for insertion. The shiny finish was seen as more slick, potentially contributing to the anticipation of a greater likelihood of being slippery and dropped during insertion, and/or involuntary expulsion from the vagina.

In addition to anticipations with respect to the finish of the ring, participants saw the surface appearance of a ring as indicative of porousness. Matte and opaque surfaces were viewed as more porous than shiny surfaces. Notably, porousness was also associated with the effectiveness of drug delivery by some participants. For some women, the greater the porosity, the better the drug delivery, thus the greater perceived product efficacy.

### Tactile Sensations of Ring Surface

Participants’ perceptions of tactile sensations associated with ring materials ranged from textured to smooth. Textured tactile sensations, like matte visual perceptions, were seen by the participants as providing an easier grip of the ring that would permit a more effective squeeze of the ring in preparation for insertion. Similar to the matte appearance, participants noted that the textured surface felt more porous.

Smooth tactile sensations elicited discussion about a smoother ring seeming more “industrial,” whereas the textured surface seemed more consistent with a medical purpose among some participants. The smooth tactile sensations did warrant some discussion about how a smoother surface would allow for less resistance in movement either along the vaginal canal or when the penis would glide across the surface of the ring.

### Material Properties

One of the main conversations within the focus group discussions was the perceptions of material “hardness” versus “softness.” The facilitators did not suggest evaluative strategies for determining hardness or softness, but the spontaneous approach by many participants was what several noted as “the fingernail test,” pressing their fingernail into the cylinder material to consider 1) how difficult it was to create a dent in the material and, 2) how long it took for the material to return to its original (rather than dented) surface form. The softer ring material (i.e., rings made of EG-85A) engendered discussion consistent with the surface appearance discussion about ease of insertion: that is, the softer ring material was perceived as more pliable and easier to squeeze.

Beyond the anticipations of insertion experiences, “softness” also suggested to the participants that the ring would be more comfortable and less noticeable when seated in the vagina. This was in contrast to those rings which were perceived as harder, as participants noted that they would expect a harder ring to feel stiff inside their vagina. They anticipated being aware of the harder material and, further, anticipated that, as opposed to the softer ring material, the harder material would not move and consequently feel like a barrier during sex.

### Dimensionality: Diameter of Cylinder and Ring

There were two important parameters with respect to the dimension of size: cylinder diameter and the overall ring diameter/circumference. Overall ring diameter had implications for participants’ initial impressions, as well as performance as sustained released products (see below); however, cylinder diameter was discussed with greatest interest, and across multiple parameters of use. A smaller cylinder diameter (i.e., 4 mm) was perceived as more comfortable, and easier to handle for insertion. Participants noted that a larger cylinder diameter (i.e., 5 mm) could pose a potential problem for their sexual partners, and expressed concerned that a larger cylinder diameter would be very noticeable and may obstruct the penis during intercourse, potentially causing discomfort to the sensitive underside of the penis. Overall, when it came to size, comfort seemed to be the main consideration when anticipating user experiences, both in daily use and during sex.

### The Interaction of Materials and Dimensionality

Participant evaluations of material properties often interacted with other properties and characteristics in their discussions of salient ring use parameters. In particular, material properties interacted with perceptions of cylinder diameter, as well as overall ring diameter, to create anticipations regarding ring pliability and flexibility, with important considerations for the insertion experience.

**Fig 1:**
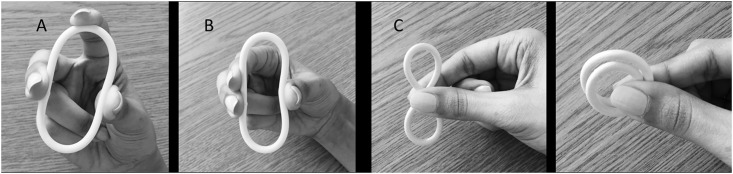
Variations in participant folding/squeezing techniques for IVR insertion. Panel A: standard squeezing of IVR involving squeezing the ring between the thumb and middle finger and using the index finger to guide, then push the ring further into the vagina. Panel B: squeezing of IVR using both the index and middle finger on one side and the thumb on the opposite side, for initial insertion; then using fingers to push the ring into place in the vagina. Panel C: the “figure-8” folding method involving squeezing the ring in the middle, twisting it at the center to make a figure-8, then folding it over, and holding the ends together for insertion.

To return to the discussion of size, comfort seemed the primary concern when efficacy was anticipated as equivalent across prototype options. Participants in at least two groups agreed that developers could make the overall diameter of the ring bigger if the cylinder diameter was small and the material was soft. Indeed, material properties interacted with cylinder diameter with respect to perceptions of flexibility. Thus, a smaller cylinder diameter using the softer of the materials made for a more flexible ring. A flexible ring was seen as one that would be pliable and easier to squeeze, making the insertion experience tolerable, if not inconsequential. A flexible ring was also seen as more comfortable, conforming to the body and staying in place better than a rigid ring. Flexibility was a particularly important characteristic for those women who preferred to insert their contraceptive ring in a folded-over figure-8 shape (see Fig 1).

Flexibility allowed the fold to open and the twist to come undone, something some participants said they could feel happening in the vagina on insertion. The harder material, especially when combined with a larger cylinder diameter, gave the impression of a ring that would be difficult to squeeze for insertion, and would result in an array of ‘insertion hold’ contortions by the participants. Some participants even experienced tiring and tightness of their fingers, in attempting to hold the ring in the squeezed position during the discussion. Further, they felt the more rigid rings would not conform to their vaginal anatomy, that they would be aware of it physically as an object in their vagina, and that it would feel unnatural, especially during sex.

In addition to issues with the force it would take to squeeze and/or hold the ring in a given position for insertion (“squeeze force”), participants also discussed recoil, labeled as “*spring back*” or “*sproing*.” These terms referred to the ring’s return to its seated shape and ‘time-to-seat’ within the vagina once they let it go during insertion. Women had particular notions about whether the ring returned to its circular shape, or took on a more oval shape, once in the vagina, as well as how quickly the ring would return to that shape once they release their squeeze. Some women reported previous experiences where they had felt their contraceptive ring “*spring”* back into place after insertion. A quick, forceful movement was not preferred by the participants, but neither was a slow movement with no detectable force. For many participants, a ring that returned to its original circular shape was perceived as one that would stay in place better. A ring that sprang back relatively quickly but not forcefully would be more appealing, as they would have the sensation that it settled into place, and would consequently feel protected. There was concern by some participants that a ring that sprang back too slowly would not settle well into a comfortable position and might slide out of the vagina if it did not return to its original circular shape.

### Implications for Sustained Release IVRs

Both material properties and dimensionality interacted when women were asked to consider ring characteristics with respect to the need for the ring to remain in the vagina for extended periods of time (i.e., from 21 to 30 to 60 to 90 to 365 days) in order to accommodate a move toward sustained release HIV prevention. In these instances, the softer material was seen as less substantive, while the harder material was seen as being more durable, and as a result, able to be effective for longer durations. Similarly, a larger cylinder diameter was appealing because of participants’ perception that it could hold greater quantities of drug, which would be required in long-term resident devices. When challenged to consider other options beyond cylinder diameter, some participants discussed the possibility of enlarging the overall diameter of the ring (rather than the cylinder diameter); and some participants, because of their concern for comfort, preferred this option.

### Conventional Acceptability Elements

In addition to the materials properties and mechanical performance of intravaginal rings that govern user sensory perceptions and how users evaluate those experiences, participants also mentioned conventional characteristics thought to impact acceptability of vaginal products, including color, ease of use, and hygiene concerns.

#### Ring Color

The majority of participants noted their preference for an opaque or transparent ring, as opposed to a ring with color. In particular, when considering the shiny appearance and smooth texture of one of the rings within the prototype set in this study, a white colored ring was evaluated negatively, and, notably by at least one participant, looked “like PVC piping.” The range for acceptability of color was discussed, proceeding from discussions about the opaque contraceptive ring either inadvertently coming out of the vagina during sex, or being purposefully removed prior to sexual intercourse: Some participants noted that, on occasion, they would have difficulty locating the ring afterward and suggested a color that allowed the ring to contrast with the surroundings and be seen.

#### Ease of Insertion and Impact of Ring Placement

While ease of use has already been discussed with respect to materials and mechanical properties, we also noted that participants reported significant variation in how providers had initially instructed them to insert the ring. Initial instructions ranged from providers stating that there was no requirement as to where the ring was to be located in the vagina, to providers stating that the ring needed to be seated encircling the cervix. Some participants expressed a lack of confidence in *both* of these scenarios. For those told that the ring could lie anywhere in the vagina, some felt initially uncertain of the ring’s efficacy when no specific placement was required. In addition, some women reported that their partners felt the contraceptive ring in their vagina during intercourse.

For those told that the ring needed to be seated encircling the cervix, their lack of confidence came from an uncertainty that they had properly placed the ring and/or that it would become dislodged in the course of their daily activities and, therefore, be less effective. To the contrary, some women believed that by placing the ring at the cervix it would be less likely that their partner would feel the ring during intercourse; thus they could leave it in at all times.

#### Hygiene Considerations

While experiences of leakage will not be as prevalent in intravaginal ring use as they can be in the use of semi-solid topical formulations such as gels, films, or tablets, participants also discussed hygiene issues related to IVR use. A few women mentioned that they felt as if the ring would become covered in vaginal secretions that they would feel the need to clean, noting how they routinely remove their contraceptive ring and rinse it off while showering to minimize vaginal fluid, discharge, and worries about vaginal odor.

## Discussion

The main goal of this investigation was to assist IVR prevention product developers in identifying the most salient user-identified product characteristics to be considered in both product design and adherence support in clinical trials and eventual real-world use. Four prototype IVRs were developed for this formative perceptibility and user experience study. We explored the relationship between product materials, dimensionality, and their interactions (e.g., pliability, flexibility) in an effort to identify and understand how these properties might impact potential users’ sensory perceptions and appraisals of use. Enrolling current and previous ring users allowed participants to use their experiences with IVRs as a point of reference, and gave them an opportunity to describe the most salient aspects of IVR use and what the prototype rings might feel like in the vagina. The prototype placebo IVRs used in this study were not under investigation in any previous, ongoing, or planned clinical trials, nor were these prototypes rings used intravaginally at any point by our study participants. The four prototypes were produced, not to find an ideal or preferred product to advance to specific clinical trials, but to qualitatively explore a range of IVR parameters and the user sensory perceptions and experiences those properties might elicit. Therefore, our findings focus not on determining a single preferred product, but on the most important product characteristics and/or properties for product developers to consider when designing and evaluating candidate IVRs, and to begin to understand concerns about expectations of use that may need to be addressed with provider and patient education.

Pliability (or flexibility) was discussed at length in each of the focus groups, and was generally considered to be the most important IVR property. Pliability, as described by the participants, seemed to be a composite characteristic influenced by materials and dimensionality, had implications for ring insertion and vaginal placement, comfort during daily use, and comfort for both partners during vaginal intercourse. Within the context of these participants’ typical IVR use (i.e., hormonal contraception restricted to 21-day use followed by 7 days for menses), most, though not all, participants stated that the prototype IVRs varied in their flexibility and stiffness, and were at least somewhat less flexible, and more stiff, than the contraceptive rings they had used; this was generally regarded as a negative characteristic of the prototypes. More pliable IVRs were believed to be easier to hold on to for insertion, easier to place in the vagina, and less intrusive for daily wear, as well as for comfort and unobtrusiveness during sexual intercourse. As noted above, however, when the discussion shifted to HIV prevention, product properties such as pliability and dimensionality (see below) became more negotiable, as participants considered whether more drug volume and/or greater durability of material over longer periods of time might be required.

Perceived product efficacy is another concept that was of particular importance for women and may be directly related to some product properties and purposes. Our previous work has demonstrated that women’s willingness to use HIV prevention products may depend on the user’s perceived product efficacy [40]. In the current study, participants related the perceived porousness of the IVR to its ability to effectively deliver active drugs to the vaginal environment. Regardless of whether a shiny versus matte finish is, in fact, related to the porosity of the ring, *and* regardless of whether that porosity corresponds to appropriate drug delivery, women’s understandings may influence their willingness to initiate and maintain product use [41]. Similarly, ring size, whether in cylinder diameter or overall ring diameter, also had implications for perceived product efficacy: participants believed that the greater the ring’s capacity to hold a drug, the more likely it would be to work, and/or work for longer periods of time in the case of long-term resident products. These characteristics are under the direct control of product developers, and such information can be used to either alter product prototypes early in the design and evaluation process, or produce educational material to preemptively address user concerns.

Hygiene and other biological concerns may represent other critical components of designing effective IVRs. Some women noted that they had removed the contraceptive ring and rinsed it during their normal showering/bathing routines. This is in line with research from the International Partnership for Microbicides indicating that between 4% and 18% of women reporting removing or expulsing the ring with many of them reporting removing the ring during menses or for periodic cleaning [42]. While women in our study had experience only with 21-day on/7-day off cycle IVRs, it may be especially important to consider hygiene concerns for HIV/STI prevention rings and MPTs as ring residency in the body advances beyond the traditional 28-day cycle. It was also noted during the focus group discussions that sustained release IVRs might be acceptable for STI prevention, including HIV, but this was sometimes countered by those participants who preferred regular menstrual cycle bleeding, as well as those concerned with having synthetic (e.g., plastic) materials in their bodies for long periods of time[41].

The impact of IVRs on the sexual experience, and the potential compensatory behaviors of IVR users, may be another important aspect to consider in IVR design. Some study participants reported removing the contraceptive ring in the context of sexual intercourse. While it is possible to remove the hormonal contraceptive ring for a few hours and not increase one’s risk for conception, depending on the specific active pharmaceutical in an anti-HIV/STI or multipurpose prevention ring, removing the ring from the vagina for extended periods of time may have a significant and deleterious impact on product efficacy. Thus perceptibility and user experiences will be important to consider in drug delivery and device design, to allow for the least likelihood that users will feel the need to remove the ring for any reason, whether sex or hygiene.

The IVR prototypes evaluated in this formative perceptibility and user experience study were limited to four prototypes that varied on two key product characteristics: cylinder diameter and materials. Other properties such as overall ring diameter were not systematically varied in this study, although emerged spontaneously in the discussion of potential increases in cylinder diameter and/or long-term residency. As intravaginal ring design work continues, other materials, properties and dimensionalities should be considered with respect to user perceptions and experiences. Additionally, these findings represent the opinions of contraceptive IVR users in the northeastern United States. Our participants were, in general, well educated, unmarried, nulliparous, Caucasian women of reproductive age. Future MPT studies will be necessary among women in other geographical regions and cultures, to understand the range of sensory perceptions and experiences of IVRs and to represent a wider range of parity, BMI, education, income and ethnicity than the participants recruited for the current study. Additionally, future work should also address the needs of adolescent girls younger than 18 and menopausal women older than 45, as both groups can be at risk of HIV and STI infection, but will likely have differing needs based on their reproductive biology and needs. We note that there are no IVR studies to date showing the predictive value of *in mano* (i.e., in the hand) evaluation to intravaginal perceptions, willingness to use, or sustained consistent use. Although the participants did not evaluate the prototype IVRs intravaginally in the context of this study, this preclinical study was designed to enroll women with previous experience with a contraceptive ring, so that that experience could inform both their comparisons with respect to sensory data, and their opinions regarding those comparisons. This is in contrast to potential concerns regarding generalizability of the sample: the findings might only apply to women willing to insert and use a 28-day hormonal IVR. While using an IVR-experienced sample sets the possibility of familiarity bias, the goal of the study was to identify and describe the range of properties that could be experienced by actual IVR users, and hence the range of properties product developers should consider in a user-centered approach to IVR design.

Given the lack of previous research in intravaginal ring perceptibility, we remain confident that these data and insights into user perspectives are of significant importance to the field’s drug delivery system design efforts. For instance, conventional acceptability studies of IVRs—conducted in the context of clinical trials—report high user acceptability in spite of ring expulsions and interference during sex. Qualitative studies such as this one advance understanding of user experiences by elucidating users’ compensatory behaviors, such as removing rings prior to sexual intercourse to maximize comfort, or rinsing rings due to hygiene concerns. With this information in mind, developers can design a ring that either minimally interferes with sexual intercourse, or that will remain protective against HIV/STI infection even if the user removes the ring for (at least) the duration of a sexual encounter. Similarly, preclinical studies should evaluate drug bioavailability and delivery in contexts where regular rinsing for hygiene purposes is anticipated. Further, it will be important to move forward in developing validated measures of user sensory perceptions and experiences of intravaginal rings and other drug delivery systems to systematically assess the impact of users’ sensory perceptions of product properties, with the goal of developing effective biomedical HIV/STI prevention products and multipurpose prevention technologies.

The context and range of discussions in this study, beyond those unique to specific physical and mechanical ring characteristics, are worth consideration in the larger scope of intravaginal ring development. The potential for covert use and the potential to maintain or enhance sexual pleasure remain critical considerations as the field continues to strive for the development of highly effective prevention technologies. Both are multifactorial constructs that complicate interpretations of data in conventional microbicide acceptability studies occurring within the context of late-stage clinical trials. Importantly, each is inextricably related to additional psychosocial determinants of use beyond the scope of preclinical product design. In the context of preclinical perceptibility evaluation such as this study, multifactorial constructs such as covert use and sexual pleasure are, in the case of intravaginal rings, importantly governed by materials and mechanical properties. Overall, the need to balance product materials, dimensions and use parameters to optimize drug delivery *and* optimize the user experience appears as important in intravaginal ring design as we find it to be in the design of topical formulations such as gels, films and other semi-solid topical drug delivery systems. We believe that by attending to these findings, product developers can leverage good user-centered product design strategy to optimize adherence to use.
